# COVID-19 pandemic and mitigation strategies: implications for maternal and child health and nutrition

**DOI:** 10.1093/ajcn/nqaa171

**Published:** 2020-06-19

**Authors:** Nadia Akseer, Goutham Kandru, Emily C Keats, Zulfiqar A Bhutta

**Affiliations:** Centre for Global Child Health, Hospital for Sick Children, Toronto, Canada; Gates Ventures, Seattle, WA, USA; Centre for Global Child Health, Hospital for Sick Children, Toronto, Canada; Centre for Global Child Health, Hospital for Sick Children, Toronto, Canada; Center of Excellence in Women and Child Health, Aga Khan University, Karachi, Pakistan

**Keywords:** COVID-19, stunting, nutrition, interventions, children, women

## Abstract

Coronavirus disease 2019 (COVID-19) continues to ravage health and economic metrics globally, including progress in maternal and child nutrition. Although there has been focus on rising rates of childhood wasting in the short term, maternal and child undernutrition rates are also likely to increase as a consequence of COVID-19 and its impacts on poverty, coverage of essential interventions, and access to appropriate nutritious foods. Key sectors at particular risk of collapse or reduced efficiency in the wake of COVID-19 include food systems, incomes, and social protection, health care services for women and children, and services and access to clean water and sanitation. This review highlights key areas of concern for maternal and child nutrition during and in the aftermath of COVID-19 while providing strategic guidance for countries in their efforts to reduce maternal and child undernutrition. Rooted in learnings from the exemplars in Global Health's Stunting Reduction Exemplars project, we provide a set of recommendations that span investments in sectors that have sustained direct and indirect impact on nutrition. These include interventions to strengthen the food-supply chain and reducing food insecurity to assist those at immediate risk of food shortages. Other strategies could include targeted social safety net programs, payment deferrals, or tax breaks as well as suitable cash-support programs for the most vulnerable. Targeting the most marginalized households in rural populations and urban slums could be achieved through deploying community health workers and supporting women and community members. Community-led sanitation programs could be key to ensuring healthy household environments and reducing undernutrition. Additionally, several COVID-19 response measures such as contact tracing and self-isolation could also be exploited for nutrition protection. Global health and improvements in undernutrition will require governments, donors, and development partners to restrategize and reprioritize investments for the COVID-19 era, and will necessitate data-driven decision making, political will and commitment, and international unity.

## Introduction

As a highly communicable disease, coronavirus disease 2019 (COVID-19) continues to ravage the state of the world's health and economy (1). Its impact also underscores the limited progress we have made against noncommunicable diseases (NCDs). Children and adults with underlying comorbidities, particularly NCDs such as diabetes, hypertension, undernutrition, and overweight/obesity, are strikingly vulnerable to serious illness and death from COVID-19 (2). Yet, COVID-19 response measures such as self-isolation, social distancing, and lockdowns of communities can lead to poor management of key risk factors such as unhealthy diets and physical activity (2), and limited access to preventive care in primary care settings. Additionally, insecure economic conditions, restricted travel and access to health care services, delayed vaccination schedules, and shuttering of educational facilities further compound poor health conditions for young children, especially in low- and middle-income countries (LMICs) (3). There is significant concern that COVID-19 responses have had a negative impact on the nutritional status of women and children, and that these could worsen over time. A recent modelling exercise of various estimates of the potential impact of COVID-19–related economic deterioration, food insecurity, and interruption of programs of community-based detection and management of malnutrition suggests that the prevalence of wasting could increase by 10–50% with an excess of ∼40,000–2,000,000 child deaths (4).

We believe that these projected nutrition effects of the global pandemic could well be underestimates, as they fail to take into account the potential effect on maternal nutrition, micronutrient deficiencies, and intrauterine growth as well as downstream impacts on maternal and child health programs that can impact linear growth and childhood stunting. This is unfortunate since the world has made some, albeit slow, progress in reducing childhood stunting over the last decade. Current estimates indicate that 149 million children under 5 y are stunted, a reduction from 166 million in 2012 but still far from the required global targets for progress (5). COVID-19 now threatens to halt or reverse gains even further. If unaddressed, the effects on linear growth in children and consequent stunting could be much more consequential than short-term effects of undernutrition.

## Risk Factors for Undernutrition in the Context of COVID-19

**FIGURE 1:**
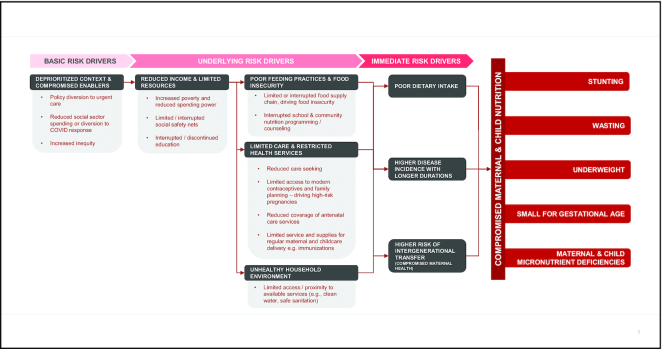
COVID-19 direct effects on basic, underlying, and immediate drivers of acute and chronic malnutrition. COVID-19, coronavirus disease 2019.

Sectors critical to reducing childhood undernutrition at particular risk of collapse or reduced efficiency due to widespread impact of COVID-19 are summarized below and in **Figure 1**.

- Food insecurity and poor-quality diets
  Building resilient food systems during COVID-19 requires innovative context-specific demand and supply-side initiatives. Food supply chains (FSCs) are of particular interest, since 80% of all foods consumed in Africa and Asia are now dependent on these markets (6). Despite being nominally “exempt” from lockdowns, COVID can have direct and indirect impacts on FSC function in LMICs, especially the informal sectors. While direct impacts, through closures of restaurants and restrictions on vendors, represent a small share of the total food economy in urban settings, the impact on rural markets could be much greater Additionally, indirect impacts due to unemployment and falling incomes of daily wage laborers and industry workers have taken a heavy toll on people in LMIC settings (7). Further compounding this is the issue of food pricing. Restrictions on mechanisms for production and delivery may drive up cost, while fear of shortages could drive speculative hoarding (8). Loss of household income exposes vulnerable families to price spikes and food shortages, while low agricultural productivity and breaks in the food import–export system disrupt local food markets and small businesses (9).
  Additionally, given limited access to fresh produce, children and families may be more likely to resort to cheaper and more accessible processed and prepackaged, high-sodium, and less-nutritious foods (10), with deleterious health consequences.
- Reduced income and limited financial resources
  COVID-19 has pushed millions of households into economic despair and has been described as more lethal than the 2008 global financial crisis (11). Oxfam predicts that half a billion people could be pushed into poverty (12), while the World Bank contends that an additional 40–60 million people could be pushed into extreme poverty (13). The interruption of existing social safety nets, especially for women, is a challenge in many LMICs struggling with COVID-19 as funds are diverted to immediate needs compounded by limited mobility and access to services.
- Limited care and restricted health services
  Given overburdened health systems, restricted travel, and changing priorities at the primary care level, access to routine health services for women and children has suffered tremendously. While quality of care was an ongoing challenge prior to COVID-19 (14), in its current state and onward for years targeted efforts for high-quality health care for those in the most need will likely take a backseat. Consequently, the health and risk of undernutrition in mothers and their children may increase dramatically, especially if current conditions persist long term. In Pakistan, available data from district health systems indicate a dramatic drop in access for and provision of antenatal care services (ZA Bhutta, personal communication, 2020), and others have highlighted the importance of the unmet need for mental health services and interventions (15). As has been indicated by United Nations Population Fund (UNFPA) (15), reduced access to family-planning services and enforced confinement of families is projected to lead to 7 million unintended births in some of the poorest countries of the world. Persistent disruptions to routine and requisite maternal care and nutrition could lead to adverse fetal outcomes including preterm birth, low birth weight, and small-for-gestational-age newborns.
- Interrupted education for children and adults
  Educational facilities, including primary, secondary, postsecondary, and specialized training institutions, have been shuttered almost completely worldwide in the wake of COVID-19 (16, 17). One of the major effects of COVID-19 has been on exacerbating inequities in education. Much has been made of alternative forms of learning, such as online classrooms, web-based courses, and home-schooling, but these are inaccessible to most children in LMICs. Women and girls, who often experience the highest rates of illiteracy and school drop-outs in LMICs, are yet further debilitated and disadvantaged. The benefits of general and specialized health and nutrition education to improve maternal nutrition and reducing intergenerational childhood stunting are indisputable, having been shown consistently in stunting case studies (18–20). An additional setback has been the interruption of school nutrition programs, the mainstay of addressing food insecurity in some of the poorest sections of the population.
- Unhealthy household environment
  Given diverted funds and priorities, building safe and healthy household and community environments, particularly as related to clean water, appropriate sanitation, and hygiene (WASH), may fall behind on country agendas. Yet now more than ever, WASH interventions are essential to protecting human health and preventing undernutrition (21). For instance, in urban slums (some of the most vulnerable communities), lockdowns and limited mobility have impacted access to clean water and safe sanitation services. Given the nature of COVID-19 transmission, this could result in lethal outbreaks of infectious diseases.

We believe that countries can address these extraordinary nutrition risks across the continuum of mothers, newborns, children, and adolescents by addressing determinants and implementing evidence-informed strategies for action.

**FIGURE 2:**
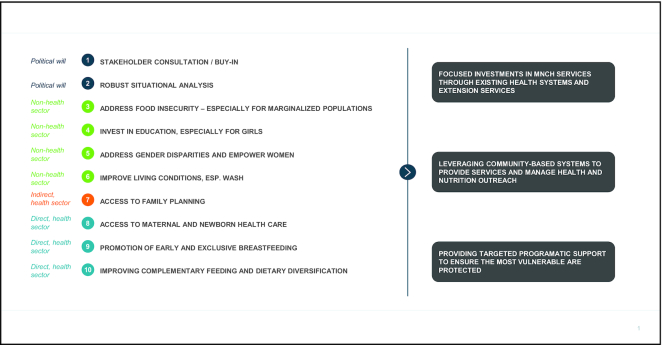
Investments to prioritize both within and beyond the health sector to mitigate COVID-19 consequences on nutrition. COVID-19, coronavirus disease 2019; MNCH, maternal and child health. Adapted from reference 22 with permission.

This narrative is aimed at reviewing key areas of concern for supporting maternal and child nutrition progress during and in the aftermath of COVID-19, while providing strategic guidance for countries to continue making headway in reducing maternal and child undernutrition while battling COVID-19. As our research into stunting reduction exemplars has demonstrated, stunting progress in LMICs has been driven by a multifactorial set of investments in sectors that have direct and indirect impacts on nutrition (**Figure 2**), most of which are extremely relevant in the current COVID-19 crisis and must be continued.

## Way Forward

Our exemplars underscore multiple examples of high-impact strategies both within and outside a country's traditional health system. These examples were data-driven and enabled by strong, focused country leadership, efficient financing, and effective partnerships (22). We believe that the same approach is needed within LMICs to address the nutritional consequences of COVID-19 mitigation strategies.

The state of the world and our collective response to COVID-19 is continually evolving as new information is received. Nevertheless, initial observations across different countries and contexts, along with key lessons from countries managing through other crises in the past, suggest that we prioritize the following approaches to address and prevent exacerbating maternal and child undernutrition:

- Food insecurity interventions
  Given the diversity of food environment and security challenges experienced by LMICs during COVID-19, solutions must be context specific. Lessons from many stunting-reduction exemplar countries could be useful. In the Kyrgyz Republic, for instance, the unprecedented economic collapse after the dissolution of the Soviet Union created new opportunities for mobilizing the agricultural sector to drive economic recovery. A range of radical agrarian reforms focused on revitalizing institutions for land, livestock, capital, and labor, while concurrently, shifting land ownership from the state to private households was considered among the most pivotal driving factors of stunting reduction in Kyrgyz Republic between 1990 and 2014 (23). While agrarian land reforms focused on shifting land ownership and adopting innovative/efficient agricultural practices may yield dividends on undernutrition in the long term, immediate solutions also have value. One of Ethiopia's solutions to food insecurity (i.e., the Productive Safety Net Program) was aimed at providing emergency food aid to 15 million individuals vulnerable to food insecurity and was considered important to the country's stunting-reduction narrative (18). Such long- and short-term solutions addressing both supply and demand-side challenges could be considered for nutrition protection in COVID-19–affected countries.
- Social protection programs
  The prioritization of efforts to provide economic security by governments to their at-risk populations (e.g., through innovative and targeted social safety net programs, payment deferrals, or tax breaks) is essential to preventing financial collapse of vulnerable households. Social-protection programs are increasingly taking center stage in policy dialogues for tackling poverty, vulnerability, and social exclusion. Several exemplar countries, notably Peru (20) and the Kyrgyz Republic (23), employed successful financial-incentive based models as a means for providing social safety nets for reaching marginalized and vulnerable populations. In Peru, for instance, the *Juntos* conditional cash transfer program provided households with a fixed monthly cash transfer (∼$30 USD) to comply with basic education, health, and nutrition services for children. This was paired with strong data-management systems that allowed for identification of vulnerable populations and effective targeting to ensure that resources were disbursed effectively. The Kyrgyz Republic's Monthly Benefit for Poor Families with Children Program is an analogous essential social-protection scheme that was found to be notably important to stunting reduction in the country. In today's COVID-19 environment, such systems in Peru, Kyrgyz Republic, and many other countries can be leveraged to build on and enhance social and economic protection for vulnerable families, and consequently prevent ill health and chronic undernutrition in children.
- Access to health care
  As has been shown in several stunting-reduction exemplar countries, access to health care for even the most remote and hard-to-reach populations can happen with an effective community health extension system. Ethiopia's health extension workers (HEWs) (18) and Nepal's female community health volunteers (FCHVs) (19) showcase successful models of mobilizing community health workers (CHWs; who receive basic training and commodities) to deliver vaccines, nutritional supplements, health and nutrition education, and even reproductive, maternal, and newborn care. The current recommendations are to remunerate such CHWs rather than rely on pure volunteerism. Amidst the COVID-19 crises, while the primary health care system may not be fully functional and supplies short, governments could consider calling on existing CHW cadres to reprioritize their tasks and cater to emerging maternal, child health, and nutrition screening in communities. These CHWs are also key to re-establishing programs for community-based management of malnutrition. Governments could also invest in deploying additional health workers and incentivizing current workers to continue delivering high-quality essential interventions to families (e.g., vaccines, antenatal care, referrals) and provide essential communication related to COVID-19 preparedness and triage. Where community health extension programs currently do not exist, countries may want to consider piloting or adopting such a program to supplement primary health care, as a short- or long-term solution.
- Educational programs
  In the wake of closed formal education systems, countries could mobilize informal institutions such as CHWs and women's and community support groups to deliver health and general education. These systems are already in place in many LMICs and could be revitalized and repurposed for continuing education. Several stunting-reduction exemplar countries have shown the potential utility and impact of these mechanisms on stunting reduction. Having learned from their experience with Ebola, Senegal's CHW program (24) has proven to be an effective mechanism for communicating health best practices to the community. Nepal's FCHV (19) and Ethiopia's HEW (18) programs have also had highly successful health and nutrition counseling components. The Kyrgyz Republic used women's support groups in communities as a means to keep updated on the evolving health situation and share knowledge (23), a model that could continue to be expanded upon.
- Safe and healthy household/community environments
  Ensuring safe water access and appropriate sanitation and hygiene practices is critical to the COVID-19 containment agenda and health outcomes beyond. While providing infrastructural support to households and communities (e.g., through building wells, community pipes, latrines) is critical, it may fall off short-term agendas as handwashing and hygiene campaigns take precedence. Lessons from exemplar countries suggest that high-impact, low-cost community mobilization efforts could play a pivotal role in creating a healthy environment by reducing open defecation and encouraging hygienic practices and have been linked to stunting reduction. The Community Led Total Sanitation (CLTS) programs in Nepal (19), Ethiopia (18), and Senegal (24) focus on behavioral change to create open-defecation–free villages. The programs trigger the community's desire for collective change through encouraging innovation and context-specific solutions while fostering a sense of community ownership. The CLTS programs in exemplar countries such as Nepal have had a notable impact on childhood stunting reduction.

Beyond interventions targeting specific challenges for childhood stunting, many ongoing COVID-19 response measures could double as opportunities to address other health and well-being priorities such as malnutrition prevention and management in LMICs. Governments, donors, and development partners during COVID-19 response policy and funding dialogues should strategize on cost and system efficiencies for targeting broader health and nutrition goals within their COVID-19 response plans.

## Conclusions

The COVID-19 pandemic has thrown the world into an unprecedented crisis, fighting a pathogen that could well be with us for a long time to come. As countries lurch from the shock of large-scale lockdowns to a gradual return to normalcy, the transition will be slow and the new normal very different from the past. Safeguarding the health and nutrition of vulnerable women and children is a key policy response and must be based on the best evidence of what works, so that gains in survival and women's and children's health and nutrition are not reversed. Governments, donors, and development partners will together need to restrategize and reprioritize investments for the COVID-19 era using data-driven decision making. Effective execution of strategies will require money, political will, and commitment, and international unity; these will be pivotal drivers, securing not only COVID-19–specific gains but also overall protection of global health and improvements in undernutrition.

## Notes

The authors reported no funding received for this study.

Data described in the manuscript, code book, and analytic code will not be made available because this is a review article and did not have primary data analysis.

Abbreviations used: CHW, community health worker; CLTS, community led total sanitation; COVID-19, coronavirus disease 2019; FCHV, female community health volunteer; FSC, food supply chain; HEW, health extension worker; LMIC, low- and middle-income country; NCD, noncommunicable disease; WASH, water, sanitation, and hygiene.
